# Impact of Coronary Endarterectomy on Mid-Term Left Ventricular Functional Recovery in Diffuse Coronary Artery Disease

**DOI:** 10.31083/RCM52886

**Published:** 2026-07-23

**Authors:** Uğur Göçen, Mehmet Aslan, Murat Yüksel, Umut Serhat Sanrı, Dolunay Odabaşı, Onur Benli, Vecih Keklik

**Affiliations:** ^1^Department of Cardiovascular Surgery, Faculty of Medicine, Toros University, 33100 Mersin, Turkey; ^2^Department of Cardiovascular Surgery, Alanya Education and Research Hospital, 07400 Antalya, Turkey; ^3^Department of Cardiovascular Surgery, Private Ortadogu Hospital, 01140 Adana, Turkey; ^4^Department of Cardiovascular Surgery, Faculty of Medicine, Alaaddin Keykubat University, 07070 Antalya, Turkey; ^5^Department of Cardiovascular Surgery, Faculty of Medicine, Çukurova University, 01790 Adana, Turkey

**Keywords:** coronary endarterectomy, coronary artery bypass, ventricular function, functional recovery, left coronary artery disease

## Abstract

**Purpose:**

Although coronary endarterectomy performed for diffuse coronary artery disease is known to increase early postoperative morbidity, its effects on mid-term myocardial functional recovery remain controversial. Therefore, this study aimed to examine changes in preoperative and sixth-month postoperative left ventricular ejection fraction, as assessed by echocardiography, in patients undergoing single or multiple coronary endarterectomy concomitantly with coronary artery bypass grafting.

**Methods:**

A total of 151 patients who underwent coronary artery bypass grafting with concomitant coronary endarterectomy, performed by experienced surgical teams between 2023 and 2025, were included in this retrospective, multicenter cohort study. Early morbidity and mortality were evaluated in the entire cohort of 151 patients, whereas the mid-term changes in echocardiographic left ventricular ejection fraction were examined in the remaining 138 patients after exclusion of the 13 patients who died in the early postoperative period. Recovery dynamics were analyzed according to the number of target vessels undergoing endarterectomy and aortic cross-clamp times.

**Results:**

Endarterectomy was performed on a total of 298 target lesions (mean 1.97 per patient) in the 151 patients included in the study, and multiple vessel endarterectomy was performed in 58.3% of the cases. The mean preoperative left ventricular ejection fraction was 46.6% ± 9.9%, and a clinically significant relative improvement of 16.8% was observed by the end of the sixth postoperative month (*p* < 0.001). Although multiple endarterectomy procedures and prolonged cross-clamp time tended to increase early morbidity, including postoperative atrial fibrillation and bleeding, no statistically significant difference was detected compared with single procedures (*p* > 0.05, *p* = 0.388)*.* In particular, complete revascularization of the left anterior descending artery territory was identified as the strongest predictor of functional recovery.

**Conclusions:**

Although coronary endarterectomy carries early risks related to increased surgical surface area and prolonged ischemia, it is associated with significant and sustained improvement in mid-term left ventricular functions among surviving patients, likely through reverse remodeling resulting from complete resolution of myocardial hibernation in the ischemic myocardium.

## 1. Introduction

In the management of ischemic heart disease, coronary artery bypass grafting (CABG) remains the primary surgical modality for improving patient survival and quality of life. Although percutaneous coronary intervention (PCI) can offer satisfactory long-term survival as a reasonable alternative to CABG, even in highly complex anatomies such as unprotected left main coronary artery (ULMCA) bifurcation lesions [1], the incidence of diffuse and extensive coronary artery disease (CAD) among patients referred for surgery is steadily increasing [2]. In coronary arteries with diffuse disease and poor distal run-off, where PCI remains anatomically inadequate, the inability to achieve complete revascularization with conventional CABG alone constitutes a major risk. In patients with complex and multi-vessel disease, incomplete revascularization leads to persistent chronic ischemia, depressed ventricular function, and an increased long-term risk of reoperation [3].

Coronary endarterectomy (CE), performed to avoid incomplete revascularization in these challenging anatomies, is a viable surgical adjunct that enables complete removal of atheromatous plaque from the vessel lumen. CE was first introduced in the 1950s for patients with diffuse and severe atherosclerotic CAD who would otherwise have been considered inoperable; as surgical techniques have evolved, CE has become a crucial adjunct in modern bypass surgery [4]. Multiple-vessel CE procedures increase the risk of early perioperative morbidity and mortality due to prolonged cardiopulmonary bypass (CPB) time, consumption of coagulation factors secondary to increased surgical surface area, myocardial ischemia-reperfusion injury, and endothelial loss. However, real-world data on the mid- to long-term recovery dynamics following maximal restoration of distal flow to the ischemic hibernating myocardium in diffuse disease remain limited.

Therefore, this study aimed to determine changes in left ventricular ejection fraction (LVEF) by comparing preoperative echocardiographic findings with postoperative findings at 6 months in patients undergoing single or aggressive multiple CE concomitantly with CABG, and to analyze the dual impact of lesion burden on early morbidity and mid-term myocardial recovery dynamics.

## 2. Materials and Methods

### 2.1 Study Population and Design

**Fig. 1.**
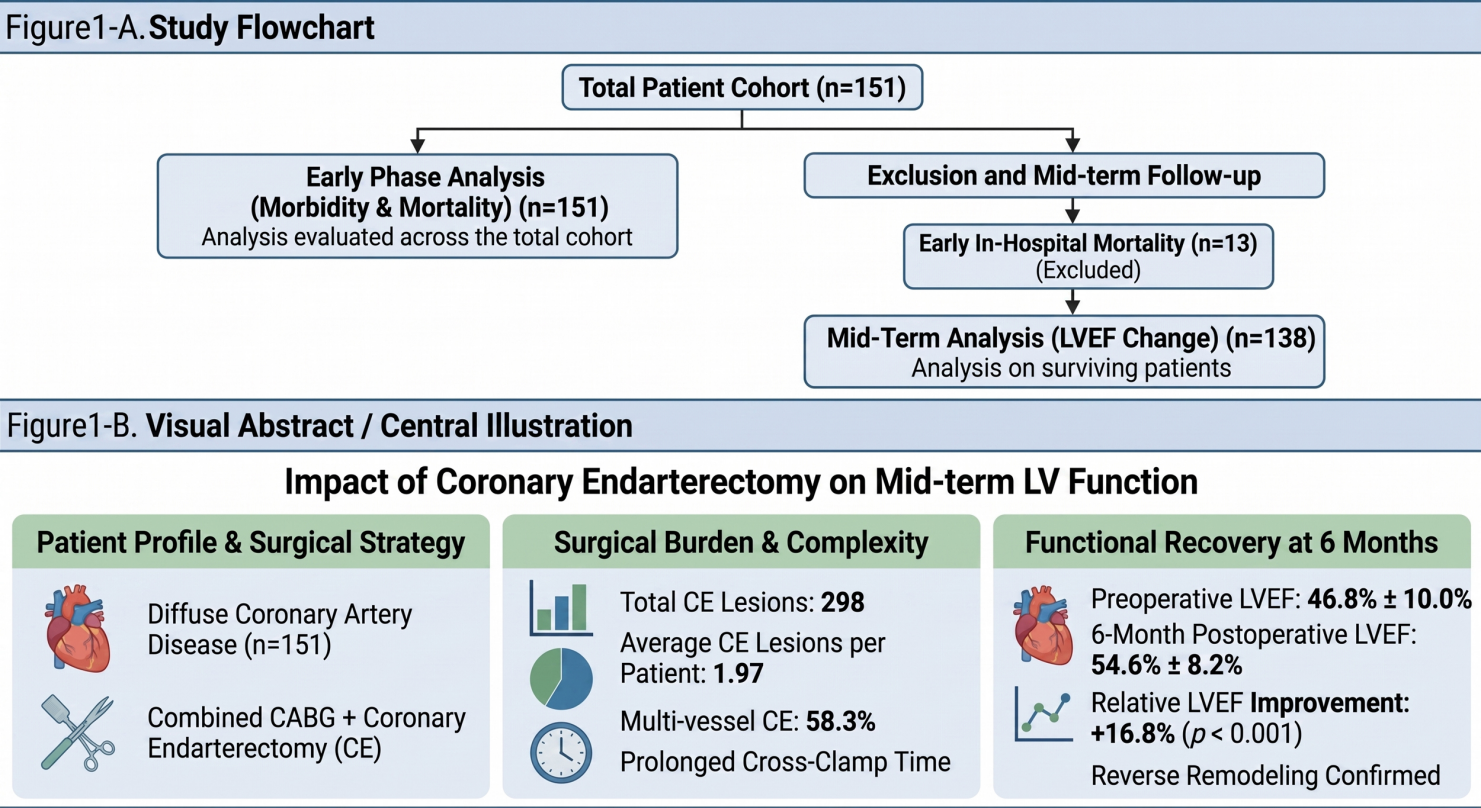
**Study Design and Clinical Outcomes**. (A) Study flowchart. Flow diagram of the inclusion and exclusion process for patients undergoing coronary artery bypass grafting (CABG) with concomitant coronary endarterectomy (CE). Early morbidity and mortality were analyzed in the entire cohort (n = 151). In contrast, mid-term changes in echocardiographic left ventricular ejection fraction (LVEF) were evaluated in the 138 surviving patients after exclusion of 13 early in-hospital deaths. (B) Visual abstract and central illustration. Impact of CE on mid-term left ventricular functional recovery (reverse remodeling) in patients with diffuse coronary artery disease. The illustration summarizes the patient profile, surgical burden (total target lesions and multi-vessel CE rate), and the significant improvement in LVEF at 6 months postoperatively (+16.8% relative increase, *p* < 0.001).

This multicenter, retrospective observational study included 151 patients who underwent CABG with concomitant CE for diffuse CAD at our clinics. Patients who underwent concomitant valve or ascending aortic surgery and those with a permanent pacemaker in the preoperative period were excluded. The study analysis was conducted in two stages: early operative data and in-hospital mortality were analyzed in the entire operated cohort (n = 151), whereas the sixth-month echocardiographic analysis evaluating mid-term functional recovery was performed in the remaining 138 surviving patients, excluding the 13 patients who died in the early postoperative period (Fig. 1A). Institutional Ethics Committee approval was obtained, and the research was conducted in accordance with the principles of the Declaration of Helsinki.

### 2.2 Echocardiographic Evaluation and Data Collection

The cardiac functional status of the patients was evaluated using the “modified Simpson method” via two-dimensional transthoracic echocardiography during the preoperative period and at the sixth postoperative month as part of outpatient follow-up. To prevent observational bias, the echocardiographic evaluations and subsequent data analyses were conducted in a double-anonymized manner; the cardiologists performing the echocardiograms and the researchers conducting the statistical analyses were completely blinded to the surgical allocation (single vs. multiple CE) of the patients. To quantitatively determine cardiac recovery, the absolute difference (ΔEF) between preoperative and postoperative LVEF values was calculated. The demographic characteristics, preoperative comorbidities, and early postoperative complications of the patients were obtained from the institutional database. Patients were divided into two main subgroups according to the number of target lesions treated: isolated/single endarterectomy (n = 1) and multiple endarterectomy (n ≥2).

**Fig. 2.**
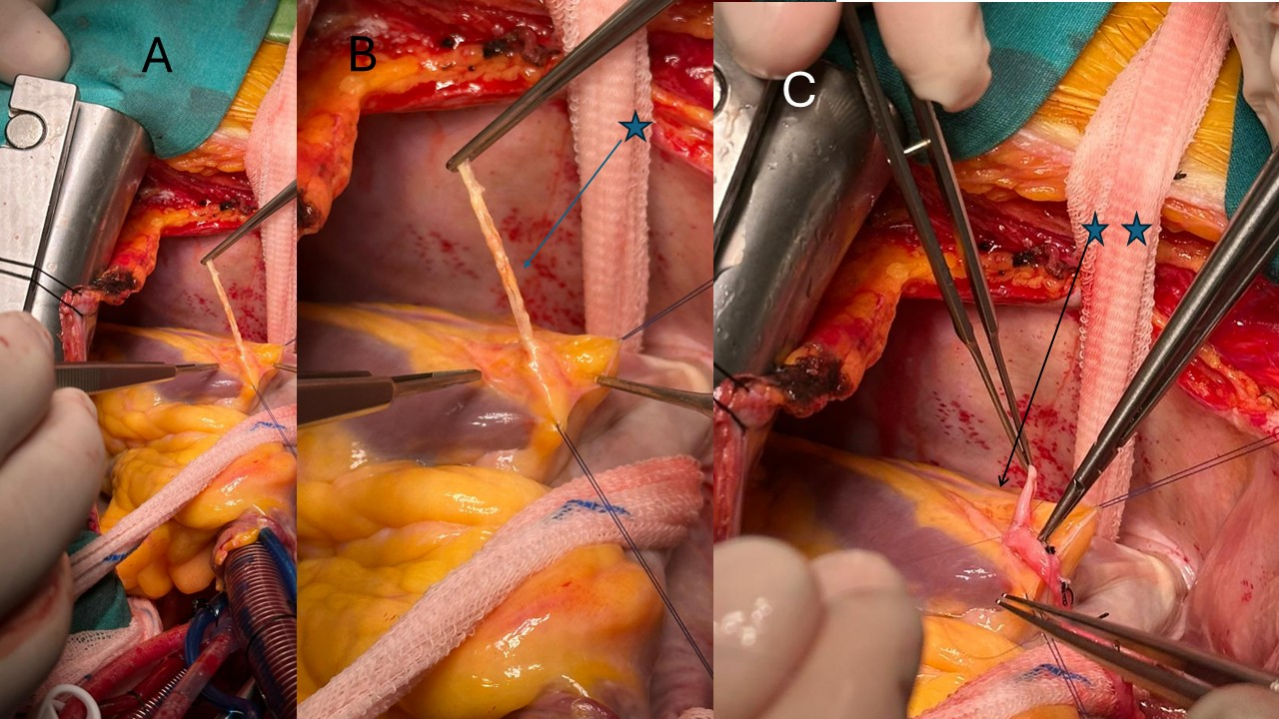
**Intraoperative surgical views**. (A) Endarterectomy performed on the right coronary artery. (B) Close-up view of the endarterectomy site; the single star (★) indicates the extracted endarterectomy material. (C) Saphenous vein anastomosis to the endarterectomized vessel; double stars (★★) indicate the anastomosis site.

Surgical technique and perioperative management: all surgical procedures were performed by highly experienced cardiovascular surgical teams. To minimize variability across centers, the surgical approaches, including the endarterectomy technique, were standardized across the participating institutions. All operations were performed via standard median sternotomy, under systemic hypothermia and CPB. CE was performed using open and closed techniques on target vessels, the left anterior descending (LAD) artery, right coronary artery (RCA), circumflex (Cx) artery, and their branches, which were considered at risk for incomplete revascularization due to diffuse plaque burden (Fig. 2; **Supplementary Video 1**). In cases of refractory bleeding that developed secondary to prolonged CPB and coagulopathy that could not be controlled medically or surgically despite massive blood product replacement, the sternum was left open and mediastinal packing (salvage hemostasis) was performed. Postoperatively, all patients received standard guideline-directed medical therapy, including optimal heart failure management and, when indicated, dual antiplatelet therapy, in accordance with institutional protocols.

### 2.3 Statistical Analysis

Data were analyzed using IBM SPSS Statistics for Windows, version 26.0 (IBM Corp., Armonk, NY, USA), and GraphPad Prism, version 9.0 (GraphPad Software, San Diego, CA, USA). Continuous variables are presented as the mean ± standard deviation (SD), and categorical variables as frequencies and percentages (%). Preoperative and postoperative changes in LVEF were evaluated using the paired-samples *t*-test, whereas differences in morbidity were evaluated using Pearson’s chi-square test or Fisher’s exact test, as appropriate. A two-sided *p*-value < 0.05 was considered statistically significant.

## 3. Results

### 3.1 Demographic and Operative Characteristics

The mean age of the 151 patients included in the study was 62.9 ± 9.1 years, and 75.5% were male. Diabetes mellitus (DM), one of the strongest predictors of myocardial dysfunction and diffuse atherosclerosis, was present in 62.9% of patients (Table 1). The mean CPB time for the entire cohort was 123.8 ± 36.4 minutes, and the mean aortic cross-clamp time was 77.4 ± 25.4 minutes (Table 2).

**Table 1.** **Baseline demographic and preoperative clinical characteristics of the study population (n = 151)**.

Variables		All patients (n = 151)
Age (years), mean ± SD		62.9 ± 9.1
Gender, n (%)		
	- Male	114 (75.5)
	- Female	37 (24.5)
Comorbidities, n (%)		
	- Diabetes mellitus (DM)	95 (62.9)
	- Hypertension (HT)	104 (68.9)
	- Smoking history	72 (47.7)
Preoperative LVEF (%), mean ± SD		46.6 ± 9.9

*Note*: Continuous variables are presented as mean ± standard deviation (SD); categorical variables are expressed as frequency and percentages n (%).

**Table 2.** **Operative parameters and anatomical characteristics of the coronary endarterectomy (CE) procedures**.

Operative variables		Values
Operative times		
	- Cardiopulmonary bypass time (min), mean ± SD	123.8 ± 36.4
	- Aortic cross-clamp time (min), mean ± SD	77.4 ± 25.4
Coronary endarterectomy lesion burden		
	- Total number of CE lesions (n)	298
	- Number of CE lesions per patient, mean ± SD	1.97 ± 1.2
Surgical strategy, n (%)		
	- Isolated single-vessel CE (1 lesion)	63 (41.7)
	- Multi-vessel CE (≥2 lesions)	88 (58.3)
Target vessel territory, n (%)		
	- CE involving LAD territory	146 (96.7)

*Note*: Data are presented as the mean ± SD for continuous variables and as numbers and percentages (n, %) for categorical variables. LAD, left anterior descending artery; min, minutes.

### 3.2 Target Vessel and Lesion Analysis

Endarterectomy was performed on a total of 298 target coronary lesions in 151 patients, with a mean of 1.97 ± 1.2 lesions per patient. Isolated single-vessel endarterectomy was performed in 41.7% of patients, whereas concomitant multiple-vessel endarterectomy was performed in the majority (58.3%). The LAD territory was included in the endarterectomy procedure in 96.7% of cases (Fig. 1B).

### 3.3 Mid-Term Functional Recovery

**Fig. 3.**
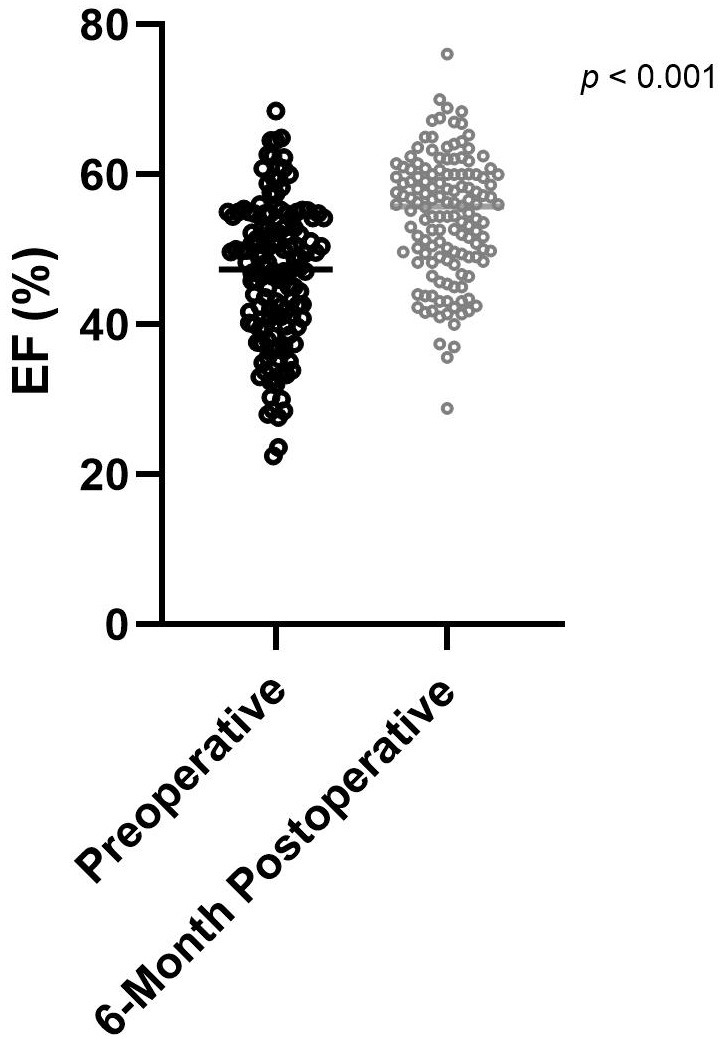
**Scatter dot plot of the individual changes in LVEF before and after CE**. The plot compares the distribution of preoperative and 6-month postoperative LVEF values. Individual measurements for the analyzed cohort (n = 138) are shown, revealing highly significant functional recovery (reverse remodeling) at the 6-month follow-up (*p* < 0.001, paired-samples *t*-test). Horizontal lines represent the mean and standard deviation.

In an internal comparison of 138 patients who completed the sixth-month echocardiographic follow-up, mean LVEF increased significantly from 46.8% ± 10.0% in the preoperative period to 54.6% ± 8.2% at the sixth-month postoperative follow-up (Fig. 3). This change indicates a clinically and statistically significant relative improvement of 16.8% compared with baseline (*p* < 0.001), with a mean absolute increase of 7.8% (95% confidence interval [CI]: 6.1%–8.9%; *p* < 0.001; Table 3). In the subgroup analysis, the increase in LVEF was quantitatively more pronounced in the group undergoing endarterectomy in the LAD territory than in the group undergoing endarterectomy in the non-LAD territory.

**Table 3.** **Early postoperative clinical outcomes and mid-term echocardiographic functional recovery at 6 months**.

Clinical and functional endpoints		Values	*p*-value
Early morbidity and mortality (analyzed in total cohort, n = 151)			
	- Postoperative atrial fibrillation (POAF), n (%)	34 (22.5)	-
	- Overall early in-hospital mortality, n (%)	13 (8.6)	-
Mid-term echocardiographic recovery (analyzed in surviving patients, n = 138)			
	- Mean preoperative LVEF (%)	46.8 ± 10.0	-
	- Mean 6-month postoperative LVEF (%)	54.6 ± 8.2	<0.001
	- Relative LVEF improvement rate (%)	+ 16.8	-
	- Vessel-specific LVEF increase (LAD vs. non-LAD)	Clinically higher in LAD	NA*

*Note*: Improvement in mid-term LVEF was evaluated using a paired-samples *t*-test. Mid-term echocardiographic recovery outcomes were analyzed after excluding 13 early mortalities. NA, not analyzed (*NA: statistical significance for vessel-specific LVEF improvement was omitted due to highly unequal sample sizes [LAD, n = 146; non-LAD, n = 5]).

### 3.4 Complications and Morbidity

**Fig. 4.**
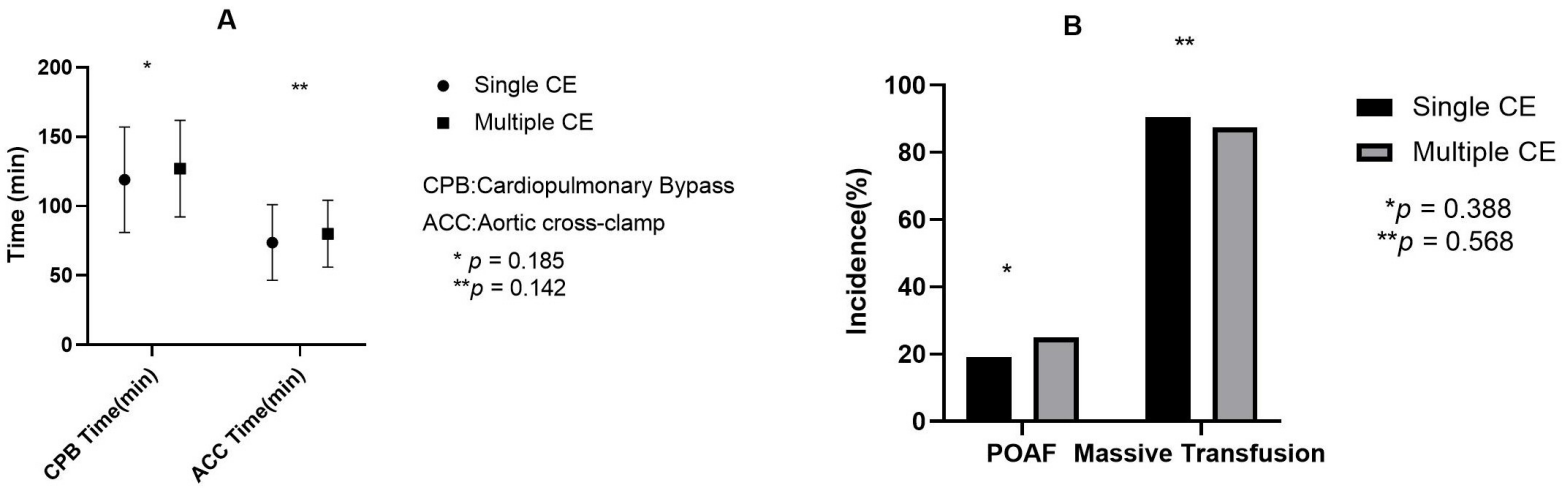
**Comparison of operative variables and early postoperative morbidity outcomes based on surgical strategy (single vs. multiple CE)**. Grouped bar chart of the impact of isolated single-vessel (n = 63) versus multi-vessel (n = 88) CE on intraoperative parameters and early clinical outcomes. (A) CPB and aortic cross-clamp times were longer in the multiple CE group, although the differences did not reach statistical significance (*p* = 0.185 and *p* = 0.142, respectively). (B) The incidence of early postoperative complications, including POAF and the need for massive blood product transfusion (≥4 units), did not differ significantly between the two surgical strategies (*p* > 0.05), indicating that an aggressive multi-vessel approach does not disproportionately increase early morbidity. CPB, cardiopulmonary bypass.*p < 0.05, **p < 0.01. No statistically significant differences were observed.

When the effect of surgical operative burden on complications was examined, CPB and cross-clamp times were longer in patients undergoing multiple endarterectomies (≥2 lesions) than in those undergoing a single endarterectomy. Although the incidence of developing postoperative atrial fibrillation (POAF) was higher in the multiple endarterectomy group (25.0% vs. 19.0%), this increase did not reach statistical significance (*p* = 0.388). Similarly, differences in the need for blood product transfusion between the groups were not statistically significant (*p* = 0.568; Fig. 4; Table 4). This finding may reflect the similarly high preoperative morbidity risk across the entire cohort, regardless of whether patients underwent single or multiple endarterectomies, due to factors such as chronic ischemia and diabetes mellitus. Despite the occurrence of POAF in 22.5% of the cohort and the complexity of the revascularization burden, the overall early in-hospital mortality rate was 8.6% (13 patients).

**Table 4.** **Comparison of operative variables and early postoperative morbidity outcomes based on surgical strategy (single vs. multiple CE)**.

Variables	Single CE group (n = 63)	Multiple CE group (n = 88)	*p*-value
CPB time (min), mean ± SD	119.1 ± 38.0	127.1 ± 34.8	0.185
Aortic cross-clamp time (min), mean ± SD	73.8 ± 27.2	80.1 ± 24.1	0.142
Chest tube drainage (mL), mean ± SD	1204.5 ± 950.2	1093.2 ± 740.1	0.439
Massive blood product transfusion, n (%)	57 (90.5)	77 (87.5)	0.568
Postoperative atrial fibrillation (POAF), n (%)	12 (19.0)	22 (25.0)	0.388

*Note*: Continuous variables were compared using the independent-samples *t*-test and are presented as the mean ± SD. Categorical variables were evaluated using the Pearson chi-square test or Fisher’s exact test, as appropriate, and are presented as n (%). Massive blood product transfusion was defined as a perioperative requirement of ≥4 units.

## 4. Discussion

This study demonstrates that CE performed in diffuse CAD ensures anatomical vessel patency and significantly improves mid-term cardiac functional reserve. Our findings suggest that myocardium that has entered a hibernating state due to chronic ischemia exhibits clear functional recovery (reverse remodeling), with a 16.8% improvement by the end of the sixth month following successful CE.

Determining the optimal revascularization strategy for the management of ischemic heart disease remains a major issue and a subject of ongoing debate. Presently, owing to technological advances in PCI, both PCI and CABG can offer similar early clinical outcomes, even in ULMCA bifurcation lesions [1]. The 5-year results of the SYNTAX II trial, published by Banning et al.[5], showed that state-of-the-art PCI strategies for *de novo* three-vessel disease yield highly promising outcomes compared with those of the past. Although this may appear to call into question the need for surgical intervention, many studies emphasize that surgical revascularization will remain relevant for the foreseeable future [1,3,6]. As emphasized by Coerkamp et al. [3], the complete elimination of myocardial ischemia and the achievement of complete revascularization in patients with chronic coronary syndromes are the most critical prognostic factors for preserving ventricular function and long-term survival. In cases of diffuse CAD with severely poor distal runoff, calcification, and extensive atheromatous plaque burden, both PCI and conventional CABG often remain inadequate. At this point, as accurately defined by Nishigawa et al. [7], CE represents one of the most important hidden weapons and valuable surgical tools in coronary surgery, used to prevent incomplete revascularization in these petrified vessels where adequate distal runoff cannot be achieved.

One of the most striking findings of our study is that the cohort predominantly consisted of multiple-vessel endarterectomy cases (mean, 1.97 lesions per patient), rather than isolated single-vessel cases. Although most studies in the literature focus on single lesions, recent real-world data reported by Bagheri and colleagues over a ten-year period demonstrate that intervention in multiple territories is often necessary to achieve complete revascularization in clinical practice [8]. However, this aggressive and extensive surgical strategy carries high perioperative costs. Lee et al. [9] highlighted the direct impact of CPB duration and surgical technique on early outcomes in CE. In our study, we observed that the postoperative recovery curve was slower when the cross-clamp time exceeded 90 minutes. At the cellular level, this clinical picture aligns closely with the pathological findings of Skeffington et al. [10], who described depletion of cardiac energy metabolites (ATP), a severe myocardial inflammatory response, and ischemia–reperfusion injury following cardioplegic arrest in open-heart surgery. The physiological insult induced by the prolonged ischemic period and the extensive endothelial debridement caused by multiple-vessel endarterectomy disrupts the coagulation cascade; this may explain the high POAF rate of 22.5%, the need for massive blood product replacement, and the use of salvage hemostatic maneuvers such as mediastinal packing observed in our study [11].

Although there has been a clear trend toward minimally invasive CABG approaches in recent years to reduce surgical morbidity, as Alsharif et al. [12] noted in their current review comparing open and minimally invasive CABG methods, the optimal surgical exposure provided by standard median sternotomy remains the indisputable gold standard for extensive endarterectomy and multiple reconstructions in diffuse disease. These perioperative costs, which increase early operative morbidity, must be accepted as an obligatory clinical trade-off to ensure functional recovery of ischemic tissue.

The most important functional finding in our subgroup analysis is that recovery, reflected by the increase in LVEF, was quantitatively much more pronounced in patients undergoing LAD endarterectomy than in those with right coronary or circumflex CAD. This robust recovery observed in our patients is consistent with the current angiographic patency literature. Costa et al. [13] reported that early arterial patency rates after CE are comparable to those of conventional bypass grafts and are satisfactory. More importantly, a 2024 study by Zahirova et al. [14] demonstrated that LIMA-LAD anastomoses performed after LAD endarterectomy achieve excellent patency rates on mid-term angiographic evaluation. Radical complete clearance of atheromatous plaque from this territory, which provides the main perfusion of the left ventricular mass, together with restoration of successful angiographic flow [15,16], likely underlies the mechanical results of the significant ventricular recovery we recorded at six months in our series.

Although the SYNTAX score, which reflects preoperative anatomical complexity, is known to directly affect long-term outcomes after standard CABG [6], our finding that this anatomical risk score was only weakly associated with late outcomes in diffuse disease LAD endarterectomy suggests that successful endarterectomy can completely neutralize anatomical disadvantages and substantially salvage the ischemic myocardium [7]. 

### Limitations

Our study has several important limitations that must be acknowledged. First, the single-arm retrospective design lacks a control group of patients undergoing CABG without endarterectomy. While we deliberately restricted our cohort to evaluate the specific clinical impact of endarterectomy, we cannot definitively distinguish the functional benefits of endarterectomy from those of standard surgical revascularization. Second, our functional analysis inherently introduces potential survivor bias. Since patients who died early could not undergo follow-up echocardiography, these individuals were necessarily excluded from the postoperative LVEF analysis; therefore, the reported functional improvement reflects only the surviving cohort. Third, due to our relatively limited sample size and event rates, we were unable to perform robust multivariable regression analyses to adjust for potential confounders, such as baseline LVEF, diabetes, or cross-clamp time, without a high risk of statistical overfitting. Consequently, independent predictors of functional recovery could not be definitively identified. Fourth, although surgical techniques and medical management were standardized according to institutional protocols, the retrospective nature of this multicenter study introduces inherent variability in center-specific practices. Finally, the assessment of myocardial viability by transthoracic echocardiography, rather than by cardiac magnetic resonance imaging (CMR), is among the main limitations of this study. However, due to its widespread availability and ease of application in routine clinical practice, echocardiographic follow-up remains the most rational and accessible method for capturing the reverse remodeling change described above. Future large-scale, multicenter, and prospective studies are necessary to address these limitations and further validate our findings.

## 5. Conclusions

Aggressive multi-vessel extensive CE performed concomitantly with CABG in patients with diffuse CAD is associated with early challenges, including POAF, the need for massive transfusion, and mediastinal packing developing secondary to extensive endothelial damage and the depletion of cardiac energy metabolites. However, despite this physiological cost, a substantial and sustained 16.8% improvement in mid-term left ventricular function can be achieved by resolving ischemic hibernation in these complex anatomies where PCI remains anatomically inadequate. Avoiding incomplete revascularization and ensuring successful reconstruction, particularly in the LAD territory that supplies the left ventricle, are critical to reversing the trajectory of heart failure. Surgeons who minimize ischemia–reperfusion injury by optimizing operative times may maximize the clinical benefit of this myocardial recovery while managing the increased surgical risks.

## Data Availability

The datasets used or analyzed during the current study are available from the corresponding author on reasonable request.
